# WHAT IS LONELINESS: INSIGHTS FROM THE BBC LONELINESS EXPERIMENT

**DOI:** 10.1093/geroni/igz038.1366

**Published:** 2019-11-08

**Authors:** Christina Victor, Pamela Qualter, Manuela Barreto

**Affiliations:** 1 Brunel University London, Uxbridge, Middlesex, United Kingdom; 2 University of Manchester, Manchester, England, United Kingdom; 3 University of Exeter, Exeter, England, United Kingdom

## Abstract

Older peoples’ views on what defines loneliness are conspicuous by their absence. The BBC Loneliness Experiment included 3 free-text questions which aimed to address this gap in our knowledge. Participants were asked to define what loneliness meant to them; their understanding of the opposite of loneliness and if loneliness could be positive and why. There were 55,000 survey responses:12,000 aged 60+. The ‘top five’ loneliness definition were: having no one to talk to; feeling disconnected from the world; feeling left out; sadness and feeling misunderstood. The most common terms used to describe the opposite of loneliness were: being connected; contentment with social relationships; happiness; friendship and availability of people. Almost 50% reported that loneliness could be positive as did 16% of those who were often/always lonely with the reasons given for this including opportunities for personal growth, the enjoyment of being alone and knowledge that the feeling would pass

